# Tunable physical properties and dye removal application of novel Chitosan Polyethylene glycol and polypyrrole/carbon black films

**DOI:** 10.1038/s41598-025-04429-y

**Published:** 2025-06-20

**Authors:** Sh. S. El-Khiyami, Heba Ali, A. M. Ismail, R. S. Hafez

**Affiliations:** 1 Physics Department, Faculty of Science, Cairo University, Cairo, 12613 Egypt; 2 Physical Chemistry Department, National Research Centre, 33 El Bohouth Street, Dokki, Giza, 12622 Egypt; 3 Spectroscopy Department, National Research Centre, 33 El Bohouth Street, Dokki, Giza, 12622 Egypt

**Keywords:** Chitosan, Polyethylene glycol, Polypyrrole/carbon black, Optical properties, Electrical properties, Dye removal, Materials science, Physics

## Abstract

In this study, polypyrrole/carbon black (PPy/C) filler with different amounts (5, 10, 15, and 20 wt%) was immobilized in a polymer blend consisting of chitosan/polyethylene glycol (CS/PEG) to produce conductive and dye adsorbent films. The study employed various distinctive techniques, including X-ray diffraction, Fourier transform infrared, and high-resolution scanning electron microscope, indicating that composites have high complexity and good interaction. Through the implementation of the UV-Vis technique, it has been observed that the reflectance of composites experiences enhancement with an increase in PPy/C content. The discussion covers the optical constants, such as the composites’ refractive index and optical conductivity. Notably, the uniform dispersion of PPy/C has caused a significant rise in the electrical conductivity of the pristine blend from 1.182 × 10^−8^ (Ω.cm)^−1^ to 1.42 × 10^−5^ (Ω.cm)^−1^ when 15% PPy/C was added. This increased conductivity is attributable to correlated barrier-hopping mechanisms. The effects of increasing PPy/C quantity, contact time (0–260 min), initial MO dye concentration (20–120 mg/L), adsorbent film dosage (0.1, 0.25, 0.5, 0.75, and 1 g/L), and the initial pH (4–10) were examined. Incorporating PPy/C up to 10% improved the removal effectiveness of the composite film. The 10% PPy/C film exhibited the maximum removal effectiveness relative to other films. Langmuir showed better conventionality than the Freundlich isotherm model with R^2^ of 0.999. The maximal adsorption capacity observed in monolayer adsorption was determined to be 217 mg/g. The adsorption of MO by the 10% PPy/C film is a chemisorption process, according to the parameters of the kinetic studies. (CS/PEG)- (PPy/C) films could be assigned to the synergistic dye adsorption effect of PPy/C filler and CS/PEG polymer-making material, ensuring excellent adsorption efficiency. Because of these appealing characteristics, PPy/C has the potential to be an environmentally friendly adsorbent in the treatment of dye wastewater.

## Introduction

The annual production of dyes is estimated to be around 7 × 10^5^ tons, and approximately 9 billion tons are dumped into water resources without treatment^1^. Because dyes are potentially harmful, their contamination is a problem worldwide. Dyes are highly noticeable in the environment due to their significant water solubility and resistance to degradation under natural conditions, resulting in a prolonged presence in the ecosystem. As a result, environmental specialists are increasingly concerned with removing colors from various harmful effluents. It is well-known that azo dyes, especially methyl orange (MO) and methyl red (MR), can cause cancer in humans. These dyes end up in aquatic environments due to their widespread use in many industries, including printing, textile, pharmaceutical, chemical, and medical labs^2^. The removal of these dyes has been investigated using various physicochemical decontamination methods, such as chemical coagulation, chemical degradation, adsorption, and precipitation. The adsorption method has garnered significant attention among other removal techniques due to its cost-effectiveness, simplicity, efficiency, reversibility, and minimal operating costs^3,4^. The study of conducting polymer-based composites for removing water-soluble organic dyes from aqueous systems has gained attention recently^5^. This article provides an update on the progress made in this research direction over the last four years. In environmental sciences, conducting polymers and their derivatives are being investigated for treating water contamination. Conductive polymers attracted particular attention because of their ease of synthesis, cost-effectiveness, readily tunable electrical conductivity, chemical reactions, biomedical uses, remarkable effectiveness in removing pollutants, high conductivity at room temperature, facile synthetic process, and environmental stability. This class of polymers exhibits significant potential for electronic devices, biosensors, and chemical sensors^6–12^. PPy has excellent adsorption capabilities, nontoxicity, environmental stability, and ease of synthesis and regeneration, and has recently become widely used for water purification^13–16^. In particular, PPy can generate several interaction mechanisms that enhance adsorption capacity and selectivity due to its abundance of –NH^+^ groups and highly delocalized conjugated system in the skeleton^15,17^. However, PPy is hindered by its low mechanical properties, brittleness, and inadequate processability. One possible solution to these shortcomings is to combine PPy with carbon black (C), improving its electrical and mechanical properties and making it more stable in various conditions^18^. And because of its high specific surface area, carbon black enhances the adsorption of dye^19^. The linear macromolecular polysaccharide known as chitosan^20–22^ has numerous applications in food, medicine, and environmental remediation^23–25^. Chitosan exhibits polycationic and hydrophilic properties due to its inclusion of hydroxyl and amino groups^23^. Chitosan offers several benefits^26^, including strong film formation, biocompatibility, biodegradability, hydrophilicity, and nontoxicity^27^. It can also treat bacterial infections and cancer and exhibits strong antimicrobial potential^28,29^. CS exhibits exceptional adsorption capabilities for eliminating dyes and metal ions from wastewater because of its large concentrations of active amino (-NH_2_) and hydroxyl (-OH) groups^30^. Consequently, adding chitosan to PPy can significantly increase its adsorption capability. However, some of its mechanical and physicochemical traits may not be sufficient for all these applications. Composite films have recently gained a lot of interest because of their higher adsorption capacity, reusability, and low production cost relative to their constituents. Combining chitosan with polyethylene glycol demonstrates a method for enhancing the mechanical properties of chitosan through blending and copolymerization^31–33^. Alexeev et al. found that combining chitosan with PEG significantly enhanced its mechanical properties^31,32^. PEG has attracted significant interest because of its excellent qualities, such as low toxicity, immunogenicity, biocompatibility, and biodegradability^34–37^. Azo dyes pose hazards to animals and aquatic life (fish, algae, bacteria, etc.) due to their genotoxicity, carcinogenicity, mutagenicity, and lethal effects. Since they are not easily broken down in the environment, conventional wastewater treatment technologies typically fail to eliminate them from wastewater. Their adverse impact on water oxygen levels and light penetration harms aquatic life. Therefore, wastewater effluent containing these dyes must be treated before being released into the environment to protect the ecological system. Our research contributes significantly to the study of the impact of the conductive polypyrrole/Carbon black composite on the structural, morphological, optical, and electrical properties of the CS/PEG blend. Adsorption processes were further investigated regarding PPy/C content, contact time, initial MO concentration, adsorbent dosage, and initial pH of the adsorption solution. To comprehend the properties of adsorption processes, kinetic models utilizing pseudo-first-order and pseudo-second-order kinetic models were examined. The novelty of this research lies in developing and investigating novel conductive composite materials, specifically (CS/PEG)-(PPy/Carbon black), of enhanced adsorption capacity for dye removal applications, easy synthesis with improved physical properties, and sustainability in wastewater treatment.

## Experimental

### Chemicals

Polyethylene glycol 6000 (PEG) and polypyrrole doped-20 wt% composite with carbon black (PPy/C) were purchased from Sigma Aldrich. Chitosan (CS) (MW = 300000) was obtained from the International Laboratory, San Francisco, USA. Methyl orange (MO) was obtained from S D Fine-Chem Limited; all these reagents are analytically pure.

### Synthesis of (CS/PEG)-(PPy/C) composite films

**Scheme 1:**
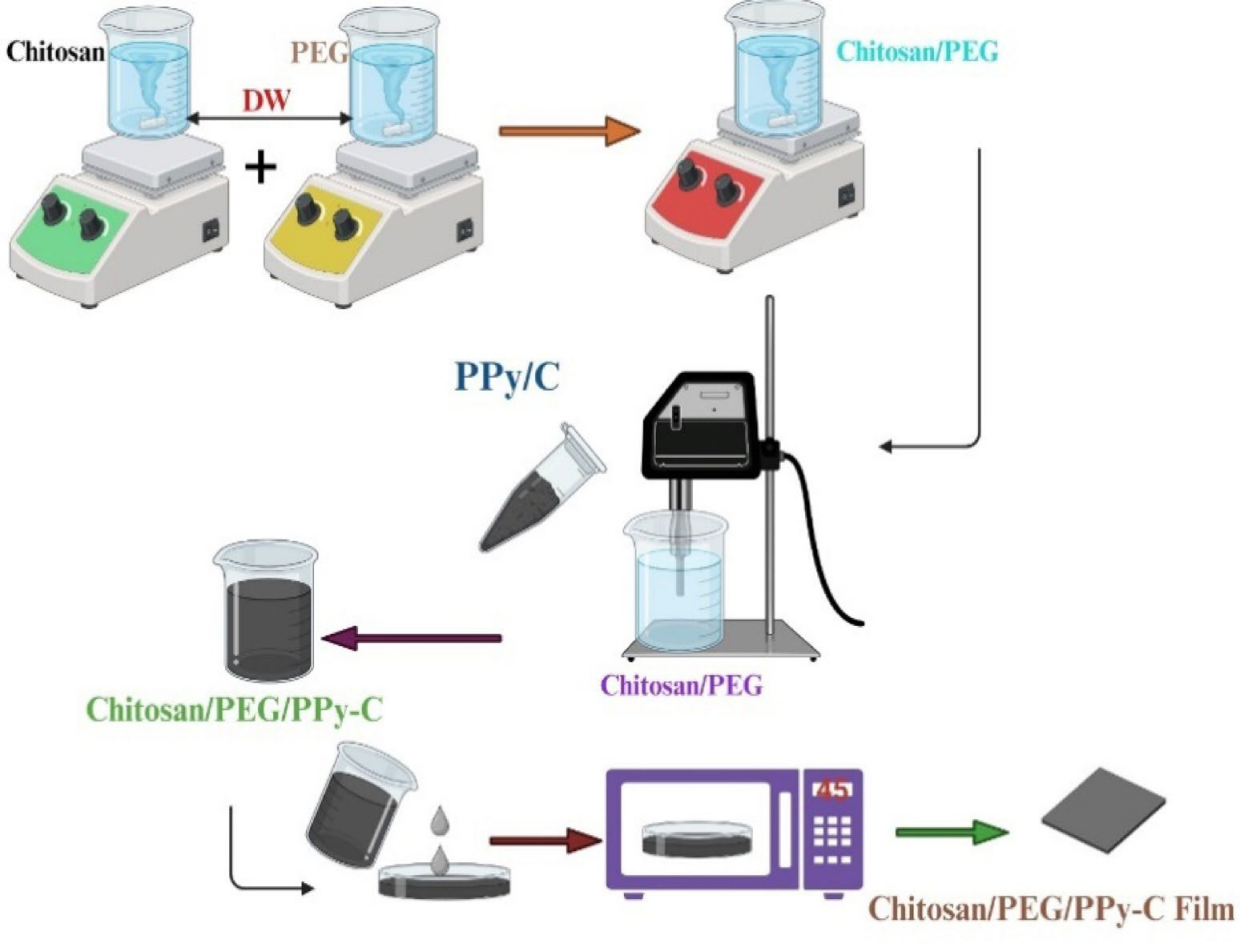
Preparation method of CS/PEG and (CS/PEG)-(PPy/C) films.

Using inexpensive chemicals to create the CS/PEG (70/30 wt%) polymer solution, 0.5 gm of CS powder was dissolved in a 2% acetic acid solution and stirred to achieve a fully dissolved and transparent solution. Subsequently, sufficient quantities of PEG were incorporated into the solution and stirred until a clear solution was achieved. The optimized ratio between CS and PEG is 0.7% w/v (weight per volume). The solution was allowed to remain undisturbed for 2 h before casting to prevent the production of air bubbles. Then, it was cast and dried in an electric oven at 45 °C for 2 days for a CS/PEG pristine sample. Following this, varying ratios (5, 10, 15, and 20 wt%) of PPy/C were introduced to the CS/PEG solution and then sonicated for 1 h. The resulting mixture was then cast into a Petri dish and dried at 45 °C for 2 days. Scheme 1 displays the preparation procedures for conductive (CS/PEG)-(PPy/C) composite films.

### Measurements

X-ray diffraction (XRD) examination was conducted using a Cu-Kα target (λ = 1.5406 Å) on an X’Pert-PRO-PANalytical apparatus, scanning over a 2θ range of 5º − 80º. Fourier transform infrared (FT-IR) measurements were performed in the 4000–400 cm^−1^ spectral band using Bruker Vertex 80 equipment. Field emission scanning electron microscopy (FESEM) from Quanta FEI, United States, was utilized to study the surface morphology of the samples. The optical characteristics of the produced films were examined using a Shimadzu UV-630 UV-VIS-NIR spectrophotometer. Dielectric measurements were conducted using Novocontrol Concept 40’s Broadband Dielectric Spectroscopy (BDS) equipment.

### Dye adsorption assessment

The dye removal activity was examined by using methyl orange (MO) dye in the presence of the modified films, including 5% PPy/C, 10% PPy/C, 15% PPy/C, and 20% PPy/C, in comparison with 0% PPy/C. The adsorption studies were conducted in a shaker at room temperature (27 °C±2). 0.02 g of the film was immersed in 40 mL of 80 ppm MO dye aqueous solution. Then, 3 mL of dye solution was taken after a specific time to measure the change in dye concentration using the Shimadzu spectrophotometer (UV-Vis 2401 PC). The influence of changing the adsorption parameters like initial dye concentration (20–120 mg/L), the adsorbent dose (0.1, 0.25, 0.5, 0.75, and 1 g/L), pH (4–10), and adsorption time up to 260 min on the decolorization efficiency was studied. After performing the studies twice, the average value was calculated.

## Results and discussion

### Structural analysis

#### X-ray diffraction (XRD)

**Fig. 1:**
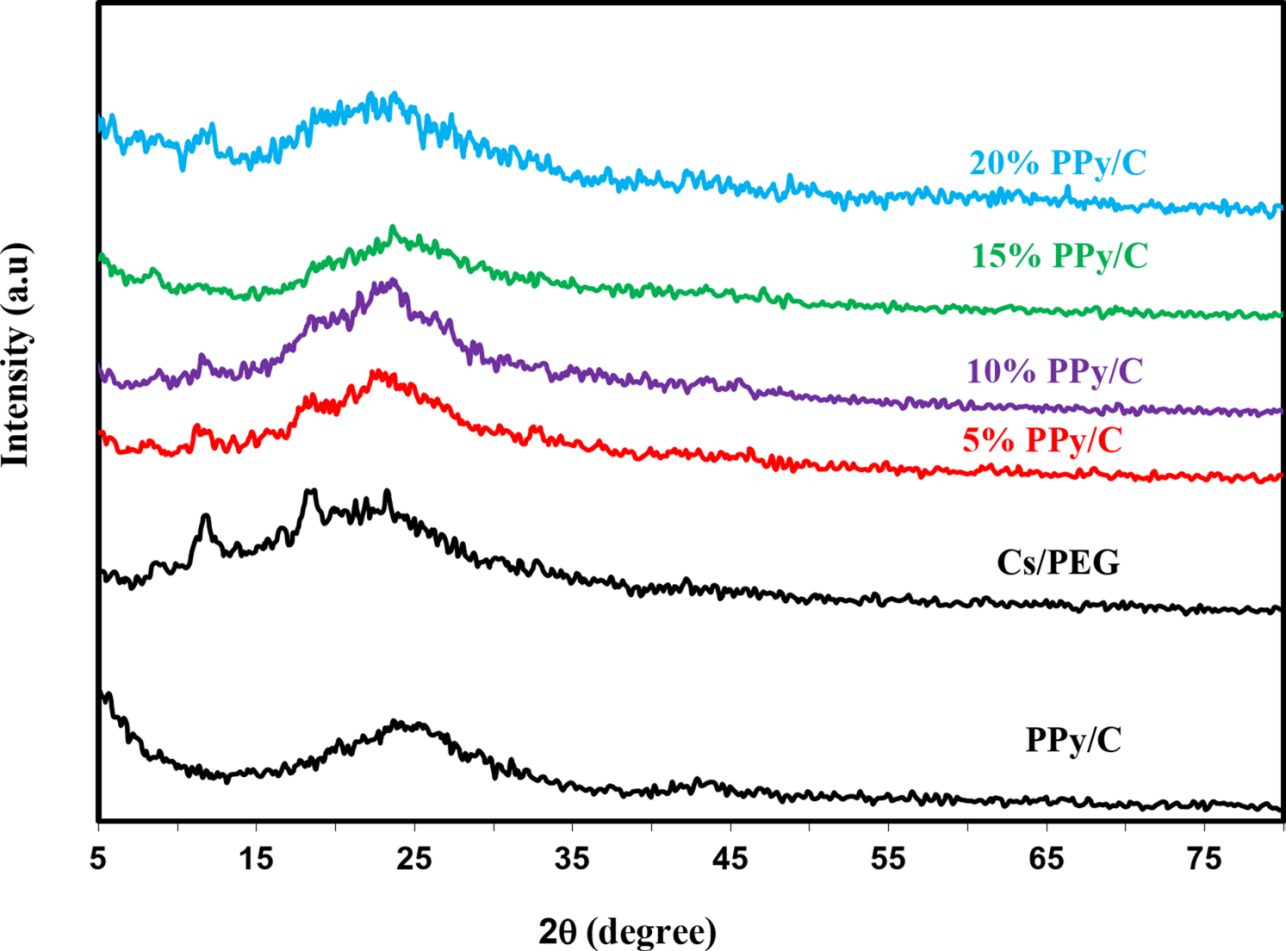
X-ray diffraction patterns of PPy/C, CS/PEG, and (CS/PEG)-(PPy/C) composite samples.

Figure 1 illustrates the XRD spectra of PPy/C, CS/PEG films with and without PPy/C composite samples. Two peaks in the PPy/C spectra were associated with PPy scattering at the interplanar spacing: a first broad peak centered at 2θ = 25.05° corresponding to a highly disordered region, proving PPy’s amorphous nature^38–41^. A second weak peak appears at 2θ = 43.85° and is attributed to amorphous carbon black^42^. The XRD pattern of the CS/PEG composite sample Fig. 1 showed relatively narrow diffraction peaks at 2θ = 12.05 °, 16.8 °, 18.81 °, and 23.37 °, which may be related to the addition of PEG to CS, indicating the compatibility and miscibility between CS and PEG^43,44^. The XRD spectra of the conductive composites (CS/PEG)-(PPy/C) showed a broad peak in the 20°- 27° region, which indicated the presence of both PPy/C and CS/PEG. As the PPy/C percentage increased to 20%wt, the peak broadening also increased and shifted toward a higher two-theta degree. The spectral diffraction peaks of CS/PEG at 2θ = 12.05º and 18.81º exhibit a decreasing intensity when the concentration of PPy/C increases. These findings indicate that combining PPy/C with CS/PEG reduces crystallinity, which can be explained by the insertion of an amorphous phase and the development of hydrogen bonds between the two matrices.

### Fourier transform infrared analysis (FTIR)

**Fig. 2:**
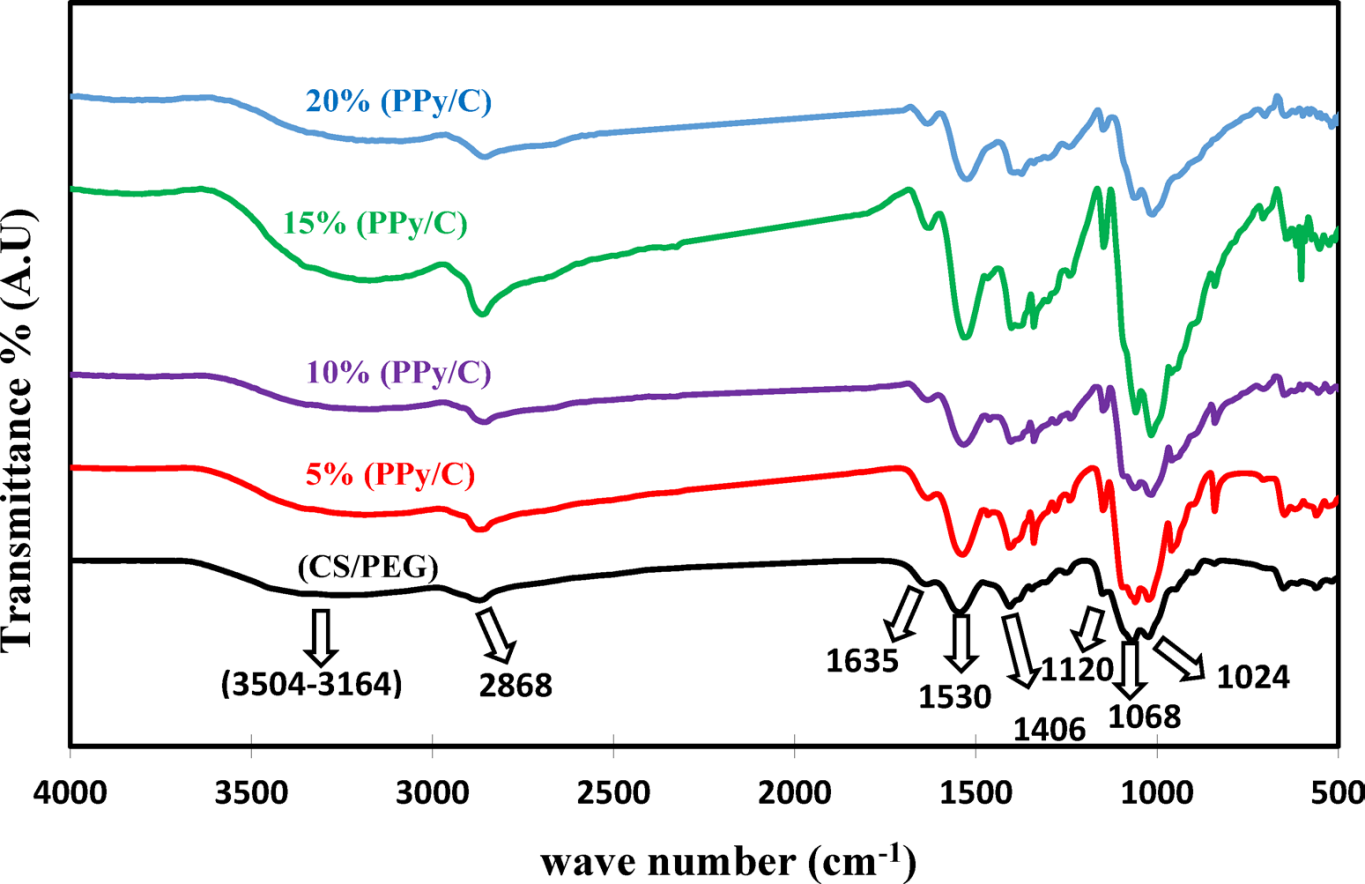
FTIR spectra of CS/PEG and (CS/PEG)-(PPy/C) composite samples.

The FTIR spectra of CS/PEG and (CS/PEG)-(PPy/C) films are displayed in Fig. 2. As expected, the CS/PEG spectrum displays a combination of CS and PEG bands, with slight variations in intensity and position^45^. In the CS/PEG spectrum, the broadband between (3504–3164 cm^−1^) corresponds to the overlapping of the OH and NH stretching vibrations^45,46^. The band shows the CH stretching mode for PEG at 2868 cm^−1^. The absorption peaks at 1635 cm^−1^ and 1530 cm^−1^ correspond to the vibrations of C = O stretching (amide I) and N-H bending (amide II). Furthermore, the C-O-C stretching vibrations of glycosidic bonds are seen at 1120 cm^−1^, 1068 cm^−1^, and 1024 cm^−1^^47,48^. Comparing the FTIR spectra of CS/PEG and (CS/PEG)-(PPy/C) films, all conductive composite films show peaks that correspond to the CS/PEG film but are more intense. As the PPy/C mass ratio increases, most of the characteristic absorption bands of composite films become more pronounced. A slight shift in some CS/PEG bands is observed in the (CS/PEG)-(PPy/C) film spectra, indicating intermolecular interactions between PPy/C and CS/PEG, likely due to newly formed hydrogen bonds.

### Field emission scanning electron microscope analysis (FESEM)

**Fig. 3:**
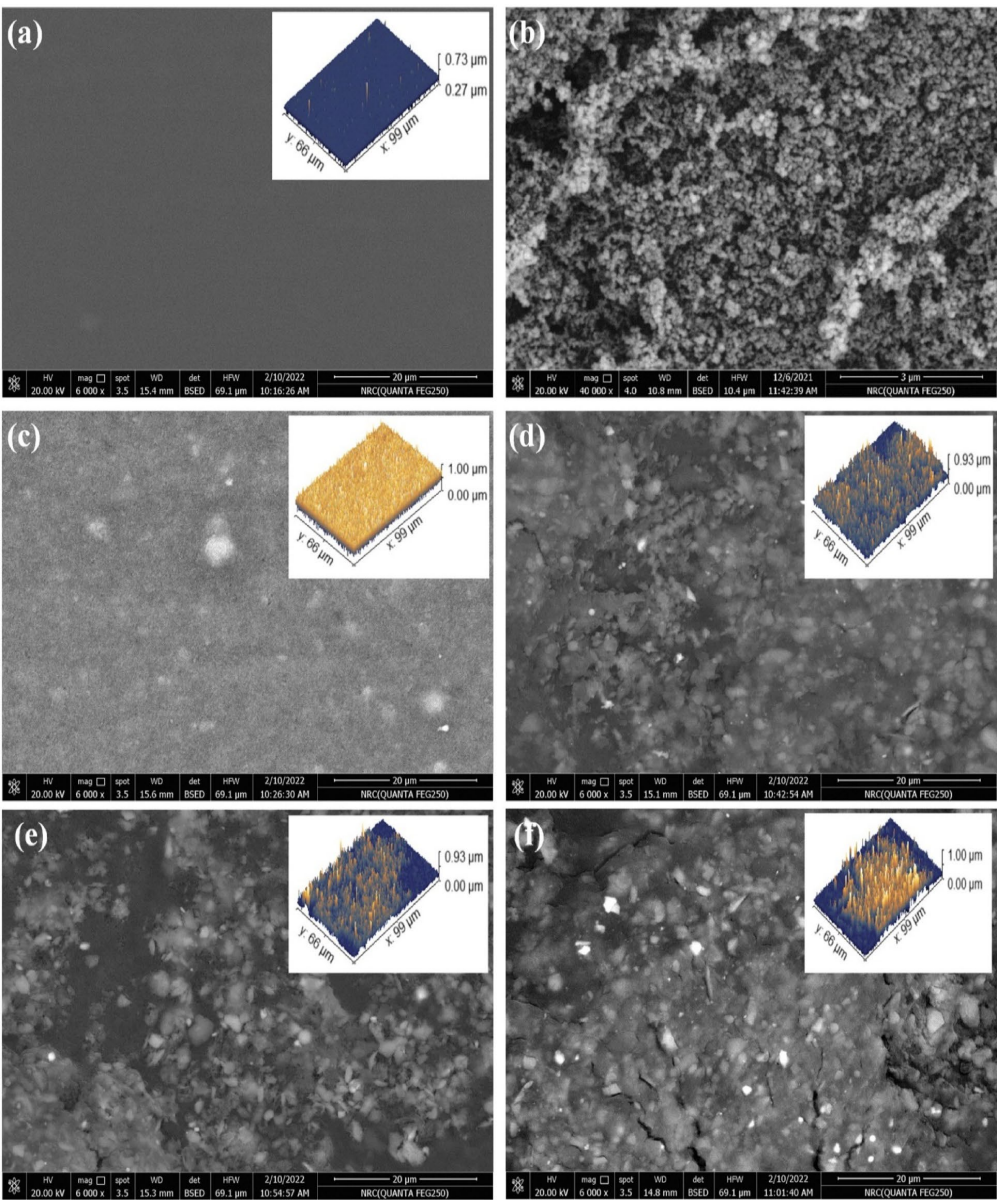
FESEM images of: (**a**) CS/PEG, (**b**) PPy/C, (**c**) 5% PPy/C, (**d**) 10% PPy/C, (**e**) 15% PPy/C, (**f**) 20% PPy/C, composite samples.

Figure 3 displays morphology investigations of PPy/C and CS/PEG undoped and doped with varying ratios of PPy/C conductive composites, which were examined using FESEM. Figure 3a shows a smooth, uniform surface, which suggests the good miscibility between CS and PEG polymers. The surface of PPy/C Fig. 3b shows a highly textured, granular surface structure with sizes ranging from 70 to 110 nm. The granular structure suggests a high surface area, which could benefit interactions with the polymer matrix as a filler. In Fig. 3c, the surface image shows the CS/PEG blend with a low concentration of PPy/C filler (5%), which appears primarily smooth with some small, bright spots scattered throughout, with small aggregates of the PPy/C filler. With increasing PPy/C, the surface shows significant texturing and numerous bright spots of various sizes. Some areas appear to have larger aggregates or clusters of the filler material^49^. Results show that the CS/PEG surface is well-coated with PPy/C, which implies that PPy/C interacts well with the CS/PEG polymer blend. The roughness of the prepared nanocomposite samples is estimated using Gwyddion software. The 3D images of (CS/PEG)-(PPy/C) polymer composites are shown in the inset of Fig. 3. The values of mean roughness (Ra) and root mean square roughness (Rt) are seen in Table 1. The findings demonstrate that the surface roughness of (CS/PEG)-(PPy/C) increases compared to the CS/PEG polymer blend. As observed in Table 1, the roughness increased in the 5% PPy/C composite, in contrast to the 10% and 15% composites. Regarding this, at low concentrations5% PPy/C, the PPy/C particles may be poorly dispersed within the polymer blend, resulting in localized aggregation and irregular surface development. With an increase in PPy/C concentration to 10% and 15%, a more uniform particle distribution is attained, facilitating enhanced integration into the polymer blend and yielding more homogeneous surfaces with reduced roughness values. The improved dispersion at these intermediate values presumably establishes a more equilibrated network of PPy/C throughout the blend. Nevertheless, the membrane of 20% PPy/C has the highest loading and displays greater roughness, possibly attributable to aggregated particles.

**Table 1:** Values of Ra and Rt of (CS/PEG)-(PPy/C) polymer composites.

Samples	Ra	Rt
(CS/PEG) blend	27.8	31.3
Blend/5% PPy/C	97.9	123.1
Blend/10% PPy/C	68.7	85.4
Blend/15% PPy/C	74.9	96.7
Blend/20% PPy/C	119.2	146.3

### UV-Visible characteristics

**Fig. 4:**
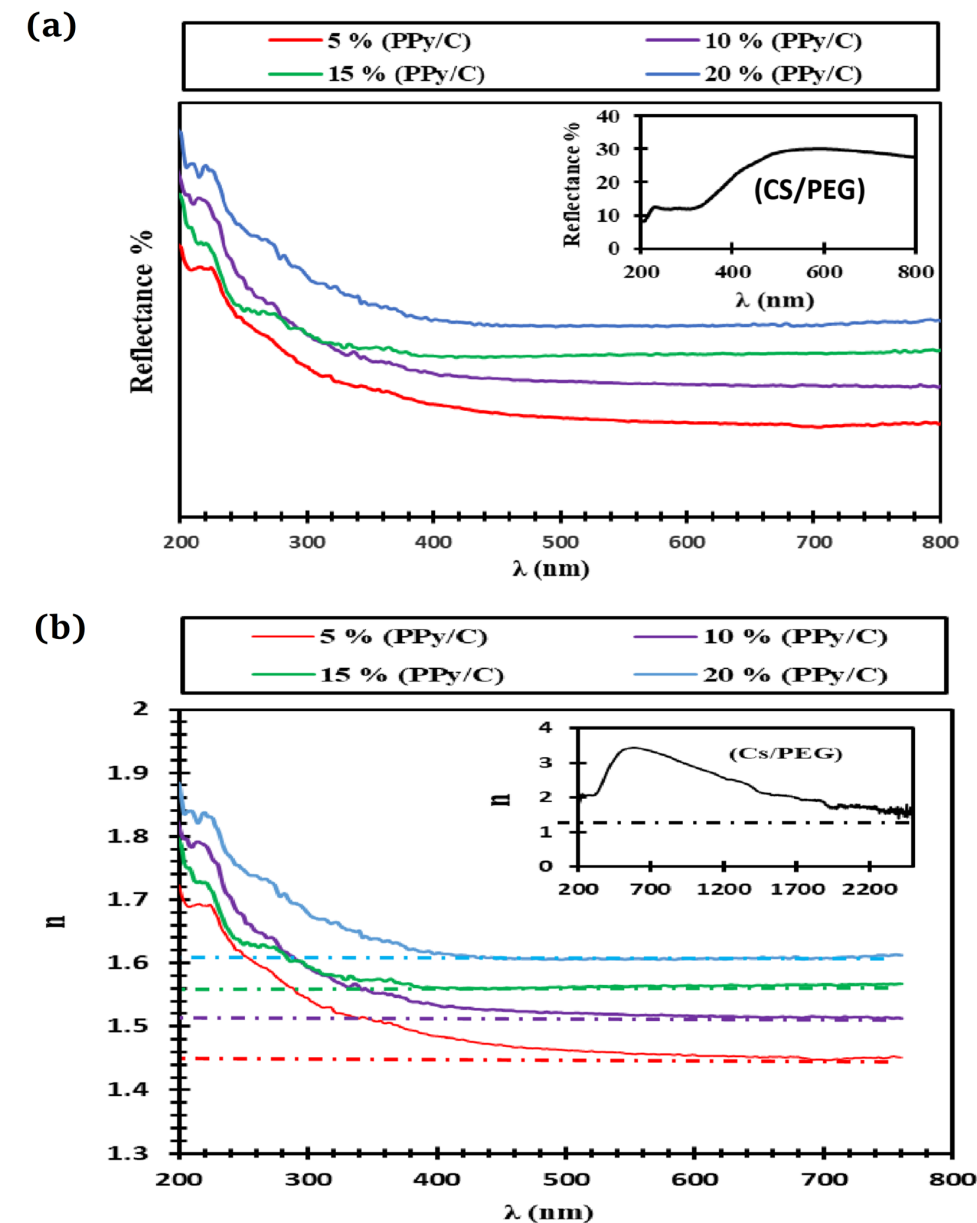
(**a**) UV-visible reflection spectra of CS/PEG and (CS/PEG)-(PPy/C) composite samples. (**b**) Refractive index (n) versus wavelength (λ) of CS/PEG and (CS/PEG)-(PPy/C) composite samples.

The diffuse reflectance spectroscopy (DRS) of (CS/PEG)-(PPy/C) films, concerning wavelength, as observed in Fig. 4a, revealed an increase in reflection values for the CS/PEG pristine film within the range of 300 nm < λ ≤ 600 nm. Furthermore, higher concentrations of PPy/C in CS/PEG led to elevated reflectance values. Increased PPy/C content can lead to higher charge carrier density, enhancing reflectance. The heightened reflectance levels indicate the films’ limited capacity to transmit light effectively. As a result, polymeric matrices doped with a high PPy/C ratio exhibit a strong ability to prevent radiation absorption within the visible spectrum. One way of calculating the refractive index (n) is by using the reflectance (R) spectra^50^:

1 $$n=\frac{1+\sqrt{R}}{1-\sqrt{R}}$$

The variations of refractive indices for CS/PEG films loaded with varying PPy/C ratios are displayed in Fig. 4b. Regarding CS/PEG, the values of n rise quickly as λ increases from 300 to 600 nm and subsequently fall as λ increases (refer to the inset of Fig. 4b). The conductive composite samples exhibit a notable decrease in refractive index as the wavelength increases, indicating the material’s usual dispersion attitude. The n value increases from approximately 1.42 for the CS/PEG film to higher values (ranging from 1.43 to 1.60) after incorporating PPy/C, suggesting that the films become denser as the refractive index rises. These elevated n values of conductive composites are encouraging and indicate that these films could be employed for strong optical confinement.

The optical conductivity (σ), which denotes the electrical conductivity resulting from the motion of charge carriers induced by the alternating electric field of incident electromagnetic waves, serves as the principal parameter for examining the optical response of materials. The optical conductivity (σ_opt_) can be computed using the following relation^51^.

2 $$\sigma_{opt}=\frac{\alpha\:nc}{4\pi\:}$$

**Fig. 5:**
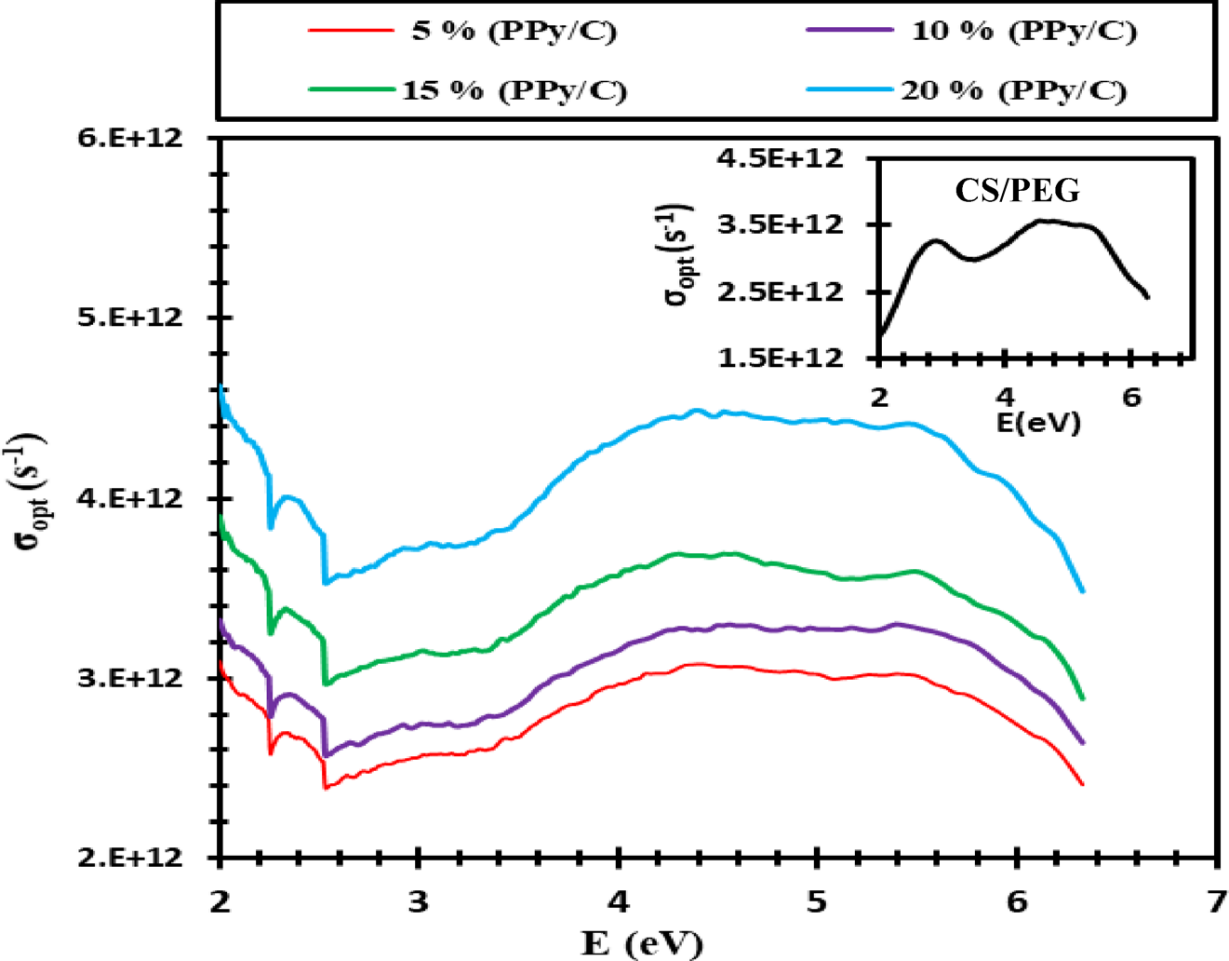
Variation of optical conductivity with photon energy for CS/PEG and (CS/PEG)-(PPy/C) composite samples.

Where C is the speed of light and α related to the absorption coefficient, the relationship between photon energy and optical conductivity for each sample under investigation is depicted in Fig. 5. At high photon energy, it is possible to see the increase in the optical conductivity of conductive composite films because of the high absorbance value in that range, attributed to the heightened charge transfer excitations. Furthermore, an increase in the optical conductivity is observed as the PPy/C content rises. This can be attributed to the amplified contribution of electron transitions between the valence and conduction bands, owing to the increased density of localized states in the band structure^52^. The prepared composite films can be used for optoelectronic applications.

### Electrical studies

#### Dielectric constant

**Fig. 6:**
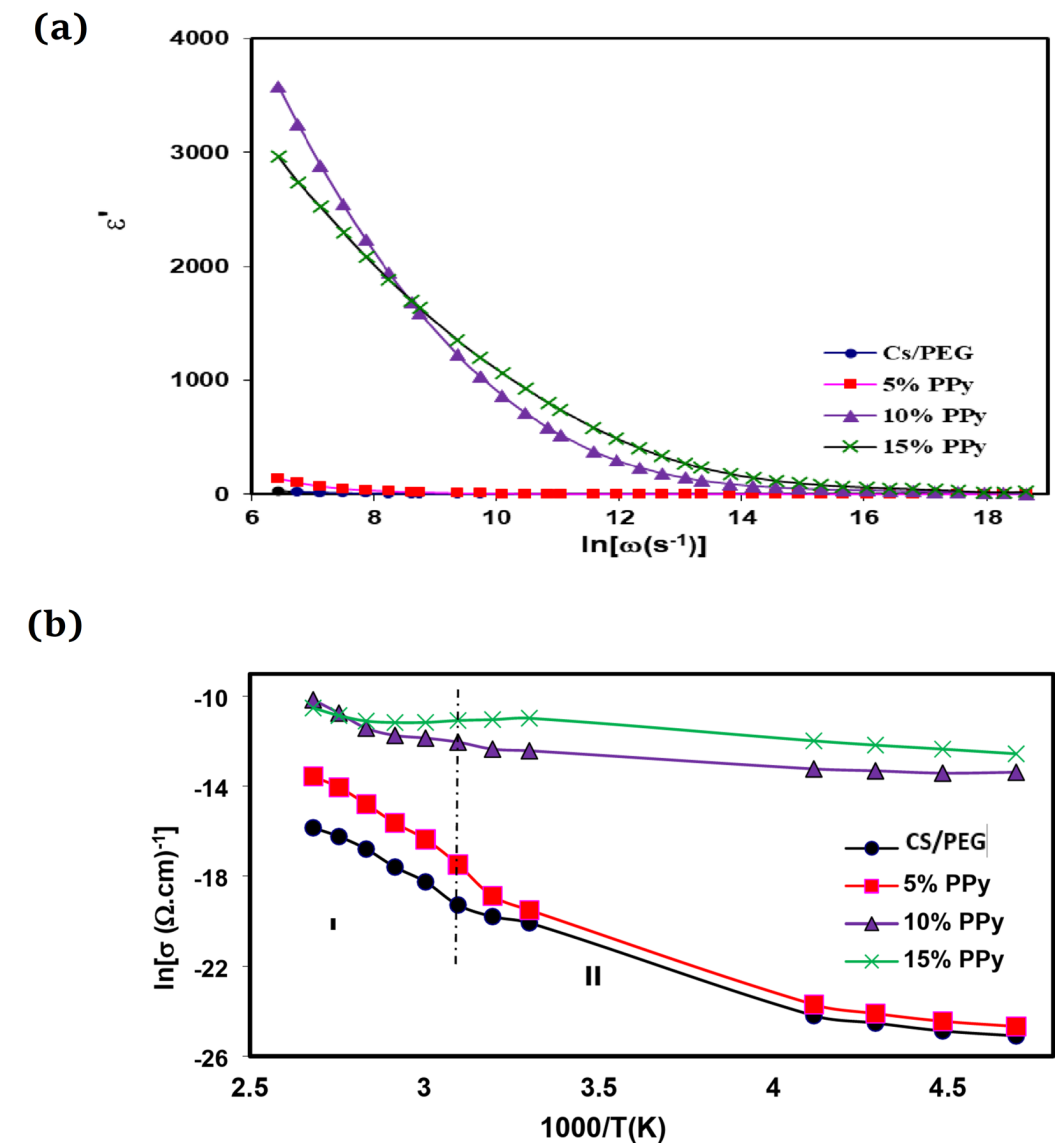
(**a**) Frequency-dependent real part of the dielectric permittivity (ε’) for CS/PEG and (CS/PEG)-(PPy/C) composite samples. (**b**) The variation of ln (σ_ac_) versus 1000/T(K) for CS/PEG and (CSPEG)-(PPy/C) composite samples at 10 kHz.

The data depicted in Fig. 6a display the variation of the dielectric constant (ε’) of CS/PEG and (CS/PEG)-(PPy/C) conductive composite films as a function of frequency. Remarkably, the initial value of ε’ is high, then as the frequency increases, the value of ε’ begins to drop, validating that for polar materials^53^. The behavior of ε’ is nearly frequency-independent at higher frequencies. The overall polarization reduces as the frequency increases due to decreased space charge polarization. This could be due to the electrical relaxation processes. However, ε’ shows a substantial frequency increase, indicating space charge polarization in the low-frequency domain. As the PPy/C concentration increases, a continuous network of conductive polymer forms inside the films, raising the ε’ of the composite samples. The ε value decreases and provides poor data at 20% PPy/C. This drop in permittivity is caused by the reduced mobility of charge carriers, primarily due to the scattering of ionized molecular aggregates.

#### AC electrical conductivity

The correlation between conductivity and temperature is a fascinating trend in materials research. Figure 6b displays the AC conductivity plot of the CS/PEG and (CS/PEG)-(PPy/C) conductive composite films vs. 1000/T(K) at 10 kHz using the following Arrhenius equation.

3 $$\sigma_{ac}=A_{0}\exp(-\triangle{E}/kT)$$

A_0_ is the pre-exponential factor, ΔE is the activation energy, k is Boltzmann’s constant, and T is the absolute temperature. The σ_ac_ plot shows that for all composite samples, the conductivity of the films increases along with temperature. When PPy/C was added, the electrical conductivity of CS/PEG film increased from 1.182 × 10^−8^ (Ω.cm)^−1^ in the blend film to 7.83 × 10^−8^, 7.07 × 10^−6^, and 1.42 × 10^−5^ (Ω.cm)^−1^ for 5%, 10%, and 15% PPy/C at 60 ^0^C, respectively. Adding conductive PPy/C to the films naturally increases their electrical conductivity, particularly at higher concentrations. However, at concentrations greater than 15%, PPy/C causes aggregation, which impedes the flow of charge carriers. The electrical conductivity rises noticeably when a small quantity of PPy/C is added. Since PPy/C is a conductive polymer and PEG and CS are nearly non-conductive, the electrical conductivity of the composite films is anticipated to rise with the addition of PPy/C. Additionally, previous works have demonstrated this development^54–57^.

Table 2 lists the calculated values of ΔE_ac_ for each composite sample. Based on the ΔE_ac_ data, it is observed that the activation energy decreases with an increase in PPy/C content. Additionally, it is found that in all composite samples, ac conduction is attributed to the hopping conduction mechanism. These findings suggest that adding PPy/C to the polymer matrix can effectively influence the ac conduction behavior of the final composite material.

**Table 2:** The dependence of the activation energy (ΔE_ac_) for CS/PEG blend on PPy/C concentration at 10 kHz.

Samples	ΔE_ac_ (eV) region I (373 K–313 K)	ΔE_ac_ (eV) region II (313 K–213 K)
(CS/PEG) blend	0.814	0.145
Blend/5% PPy/C	0.712	0.139
Blend/10% PPy/C	0.465	0.084
Blend/15% PPy/C	0.167	0.067

The frequency-dependent conductivity is commonly characterized using the universal dispersion relaxation (UDR)^58^.

4 $$\sigma_{(\omega)}=\sigma_{dc}+A\omega_{s}$$

where σ_dc_ is the dc conductivity of the sample, A is a temperature-dependent constant, and s is the power law exponent. In the hopping models, the “exponents” reflect the degree of interaction between mobile ions and their surroundings. It is typically within the range of 0 < s < 1. A thermally activated hopping process between two locations divided by an energy barrier explains the transport mechanism^59,60^.

**Fig. 7:**
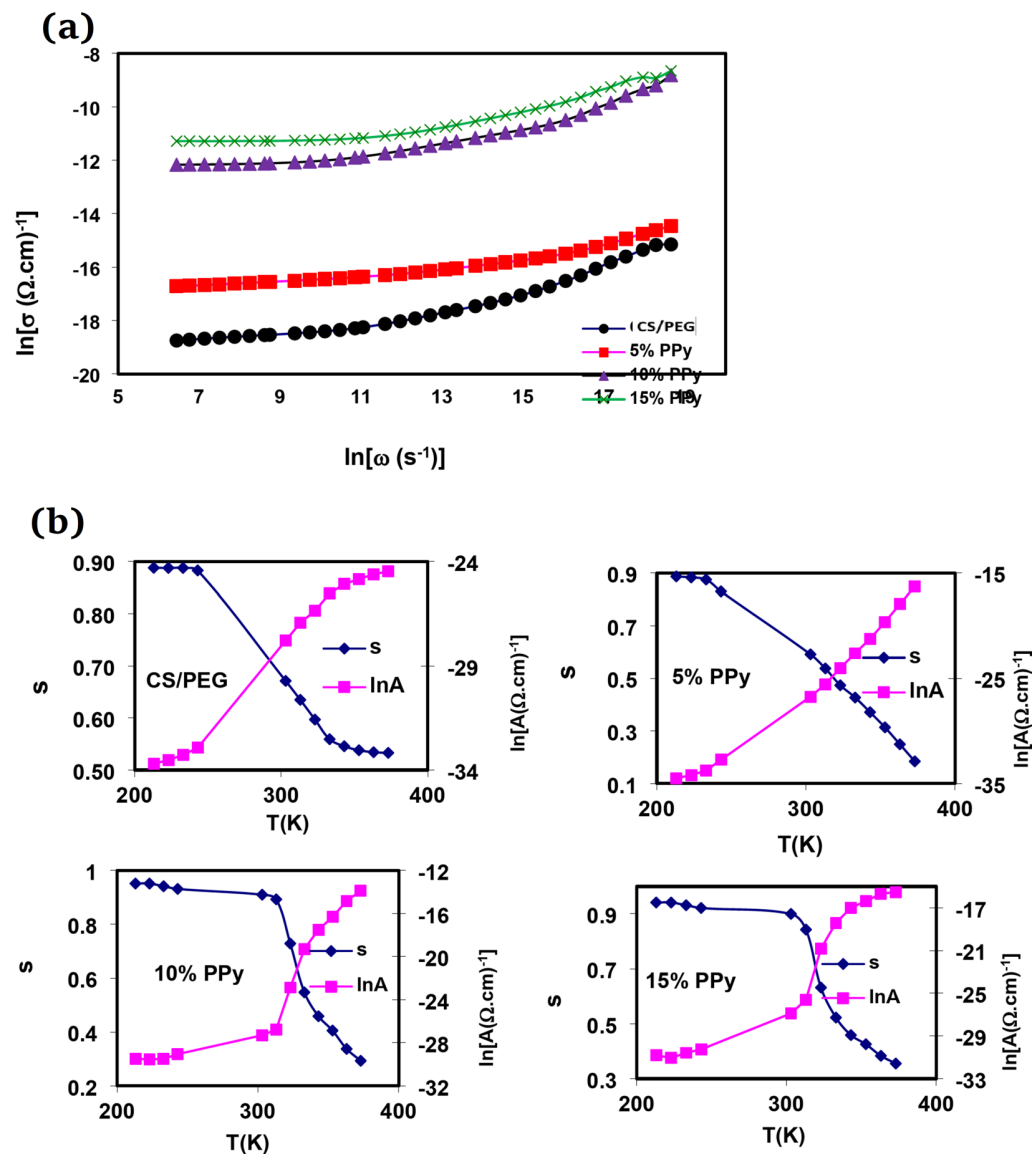
(**a**) The dependence of ln (σ_ac_) on frequency for CS/PEG and (CS/PEG)-(PPy/C) composite samples at 60 °C. (**b**) Thermal variation of factor A and exponent s for CS/PEG and (CS/PEG)-(PPy/C) composite samples.

In Fig. 7a, the logarithmic plot illustrates the relationship between the electrical conductivity (σ) of the polymer composites and the angular frequency (ω) at room temperature. The polarization of the space charge at lower frequencies and charge carrier hopping are responsible for the observed rise in electrical conductivity with frequency, as detailed in reference^61^. In Fig. 7b, we can observe how the exponent “s” varies with temperature for both the pure CS/PEG blend and the (CS/PEG)-(PPy/C) conductive composite films. As the temperature rises, the exponent “s” decreases and consistently remains between 0 and 1. These results suggest that the most suitable mechanism to explain the AC conduction behavior in all the samples studied is correlated barrier hopping (CBH), as indicated by previous research^62^.

### Dye adsorption studies

The adsorption efficiency of CS/PEG film before and after sensitization with PPy/C filler was studied using MO dye as an example of an anionic dye. Different parameters, including the incorporated amount of PPy/C (5, 10, 15, and 20 wt%), adsorption time (0–260 min), pH (4–10), adsorbent film dosage (0.1, 0.25, 0.5, and 1 g/L), and initial MO concentration (20 − 120 ppm) were studied.

#### Impact of the PPy/C content

**Fig. 8:**
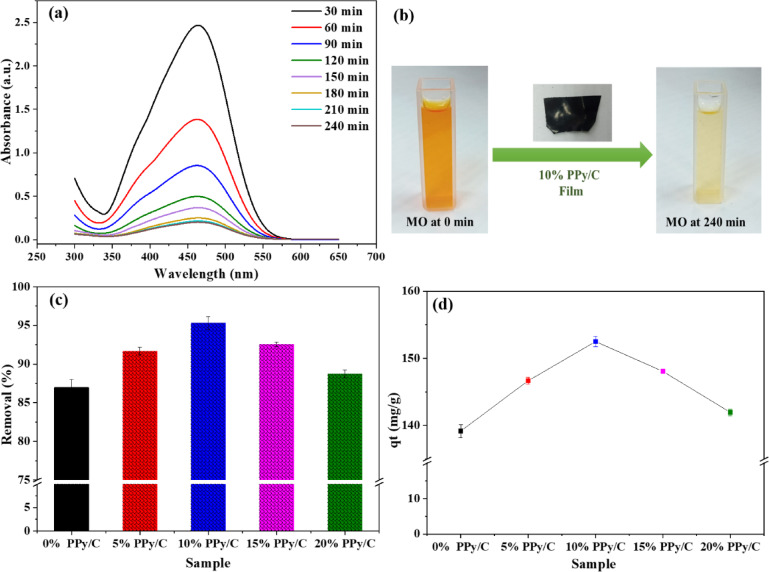
(**a**) UV-Vis absorption spectra of MO dye with 10% PPy/C film. (**b**) Digital photos for 80 ppm MO solution at 0 min and after immersion of 10% PPy/C film for 240 min. (**c**) The removal percentage for MO dye and (**d**) The adsorption capacity (q_t_) for all the prepared films. Conditions: MO concentration = 80 ppm, time = 180 min, and dose 0.5 g/L.

The CS/PEG polymer matrix was modified with different amounts of PPy/C using a solution casting process. The MO absorption spectra with a 10% PPy/C film are displayed in Fig. 8a. The dye concentration was estimated from the curve by measuring the relevant absorbance value of the MO at λ_max_ (464 nm) at various adsorption times. Figure 8b displays a digital photo of 80 ppm MO solution at 0 min and after immersion of 10% PPy/C film for 240 min. It is clear that before the immersion of the developed film, the MO dye exhibited a very intense yellow color. In contrast, after 240 min of adsorption, the dye solution became pale yellow. This indicates the adsorption abilities of the 10% PPy/C film toward the MO dye.

The dye removal percentages (R%) and the adsorption amount (q_t_) were determined using Eqs. 5 and 6, respectively.

5 $$\:\text{R}{\%}=\:\frac{{\text{C}}_{0}-{\text{C}}_{t}}{{\text{C}}_{0}}\:\times\:100\:\%$$

6 $$\:{\:\:\:\:\:\:\:\:\:\:\:\:\:\:\:\:\:\:\:\:\:\:\:\:\:\:\:\:\:\:\:\:\:\:\text{q}}_{\text{t}}=\:({\text{C}}_{0}-{\text{C}}_{\text{t}})\times\:\frac{\text{V}}{\text{m}}$$

Where C_0_ represents the concentration of MO at time t = 0, C_t_ represents the concentration at time t (in min). V represents the MO dye solution volume, and m represents the film mass. The dye decolorization efficiency of various modified films (5, 10, 15, and 20%) compared to the unmodified CS/PEG film was estimated as presented in Fig. 8c. The adsorption conditions were as follows: dosage = 0.5 g/L, C_0_ = 80 ppm, and duration = 180 min. All modified films exhibit higher removal percentages than the pristine CS/PEG film. Incorporating PPy/C improved the adsorption properties of the CS/PEG film, regardless of the amount of PPy/C added. At 180 min of adsorption time, the 10% PPy/C film exhibited the highest removal percentage relative to various films, reaching 95.3%. It was increased to 96.3% at 240 min. The adsorption capacity of the synthesized films after 180 min of adsorption was determined using Eq. 6, as illustrated in Fig. 8d. The maximum adsorption capacity recorded experimentally was 152.5 mg/g for the 10% PPy/C film at C_0_ = 80 ppm and 180 min. The obtained results suggest that the PPy/C sensitization amount increases the adsorption properties of CS/PEG until a definite value is reached, and a further rise in the amount of CS/PEG decreases the adsorption efficiency. This decline can be attributed to the agglomeration of PPy/C, which represents the active adsorption sites and reduces the adsorption performance. This performance agrees with the literature^63^. Based on these results, the 10% PPy/C film was used for further adsorption experiments.

#### Impact of adsorbent dose

**Fig. 9:**
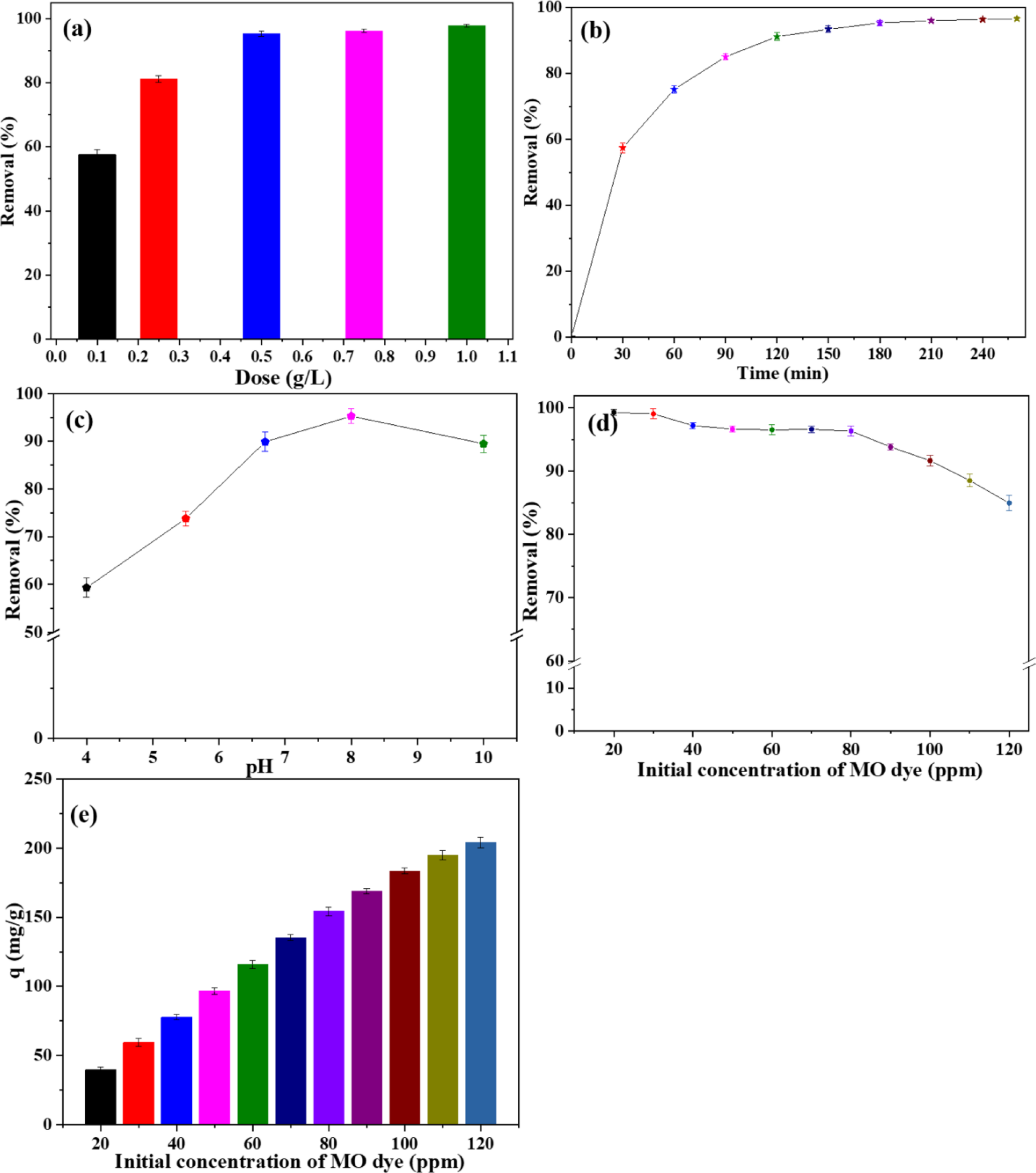
Impact of (**a**) adsorbent dose, (**b**) Contact time, (**c**) pH, (**d**) Concentration of MO dye on the removal of MO dye, and (**e**) Experimental q (mg/g) at various concentrations of MO dye in the presence of 10% PPy/C film.

To find the optimal amount of the adsorbent material to achieve maximum removal effectiveness while minimizing material waste, various doses, including 0.1, 0.25, 0.5, 0.75, and 1 g/L, were investigated using 80 ppm MO at 180 min. The elimination percentage for MO in the presence of 10% PPy/C film with different doses is shown in Fig. 9a. The data indicate a significant increase in removal percentage from 57.6 to 95.3%, with an increase in adsorbent dose from 0.1 g/L to 0.5 g/L. The removal percentage increased to 96.2% and 97.8% when the concentration was raised to 0.75 g/L and 1 g/L, respectively. These findings suggested that the appropriate adsorbent dose for the dye removal experiments is 0.5 g/L. The increase in the quantity of the adsorbent material correlated with a rise in the number of active adsorption sites, enhancing removal efficiency up to a specific threshold. Beyond this point, additional doses produced only minor changes in adsorption performance^64^.

#### Impact of contact time

Knowing the ideal adsorption time on a laboratory and industrial scale is crucial to attaining the maximum dye removal percentage. The elimination percentage for an 80 ppm MO solution is depicted in Fig. 9b in the optimal film containing 10 wt% of PPy/C. The elimination % increased gradually, reaching 95.3% when the contact time increased to 180 min. Up to 240 min and 260 min of adsorption time, the dye removal was ~ 96.3% and ~ 96.6%, respectively. This performance may be elucidated as follows. Because the sites for adsorption are empty at the start of the contact time, the adsorption effectiveness increases dramatically until the system reaches equilibrium or saturation. The percentage of removal remains relatively unchanged later. Similar results were also found in other studies^65^.

#### Impact of pH of dye solution

The sorption of dyes is significantly influenced by pH, impacting both the adsorbent’s surface charge and the adsorbate’s chemical structure. Dilute hydrochloric acid or sodium hydroxide was used to adjust the pH. The adsorption condition was 80 ppm MO solution and 180 min. As is evident from Fig. 9c, the removal of MO dye depends on the environment’s pH and was investigated in the range of 4–10. In the pH 6–8 range, the film exhibited the maximum sorption of MO dye, but the dye removal decreased under strongly acidic and alkaline conditions. The reduced adsorption capacity of MO dye by the 10% PPy/C film at lower pH levels can be attributed to the dissolution of chitosan below pH = 6, leading to decreased adsorption efficiency. The adsorption capacity increases when the pH level is neutral or above, attributed to the electrostatic interactions between the film and the anionic MO dye. However, the adsorption rapidly declines when the pH level equals 10. Two facts explain this abrupt decline. An increase in pH initially generates a significant quantity of OH^−^ ions, which compete with MO dye for binding to the functional sites. Secondly, the NH_2_ groups undergo deprotonation, leading to electrostatic repulsion between the NH^−^ ions and the anionic MO dye^66^.

#### Impact of dye concentration

Figure 9d demonstrates how the effectiveness of adsorption in the presence of 10% PPy/C film changes as the initial concentration of MO dye increases. The concentration of the MO stock solution was 200 ppm, and the range of dilutions of MO used in the study ranged from 20 to 120 ppm. Following previous research^64^, we found that a higher starting concentration of MO dye resulted in a lower decolorization %. The adsorption efficiency declines with increasing dye concentration while maintaining a constant amount of adsorbent material. Owing to using higher concentrations of dye, the adsorption time increased to 260 min to reach equilibrium. Figure 9e shows the experimental quantity adsorbed (q, mg/g) using various concentrations of MO dye in the presence of 10% PPy/C at 260 min. The optimal 10% PPy/C film exhibited a quantity adsorbed of about 203 mg/g.

### Adsorption isotherm models

**Fig. 10:**
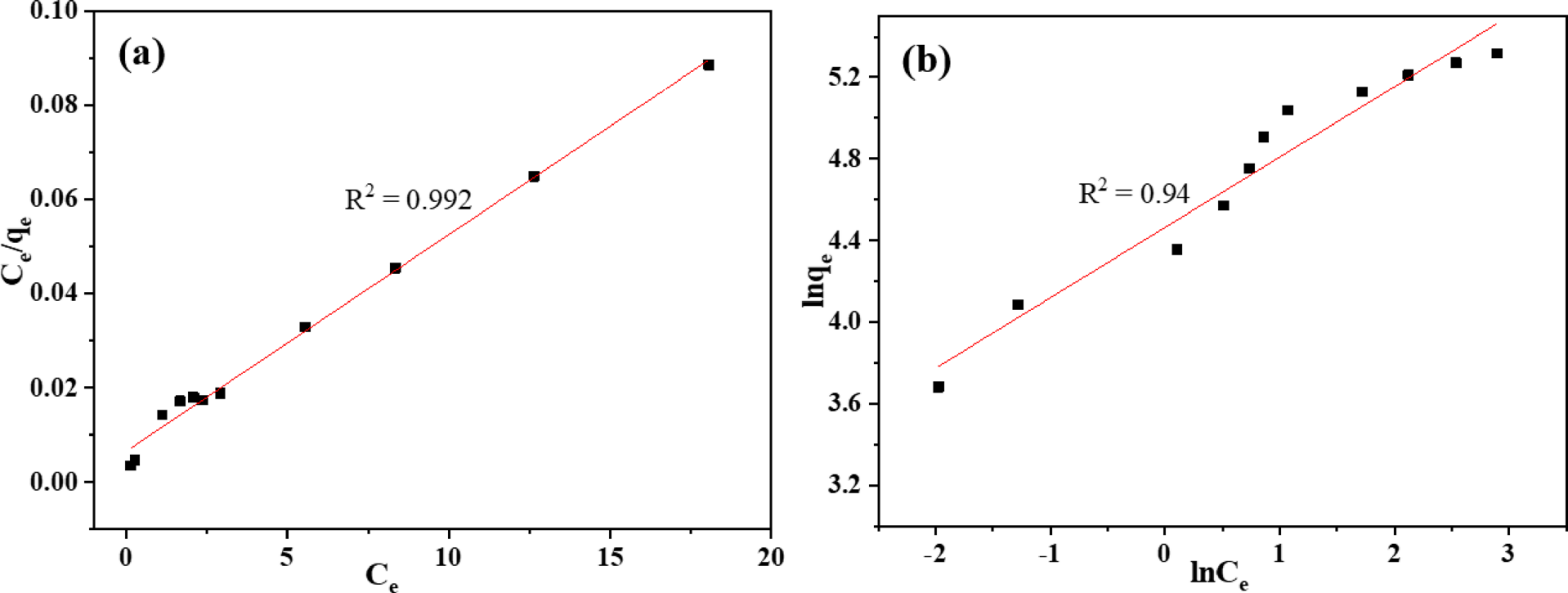
The fitting of (**a**) Langmuir and (**b**) Freundlich isotherm models for the sorption of MO dye using 10% PPy/C.

To enhance the understanding of the sorption behavior of MO dye on the 10% PPy/C film, the experimental data were analyzed using two adsorption isotherms: Langmuir and Freundlich. Figure 10 illustrates the fitting of the Langmuir and Freundlich isotherm models. Langmuir model, the adsorbent material is assumed to have a uniform monolayer coverage as each active adsorption site on its surface has an equal affinity to the adsorbate (i.e. monolayer adsorption)^67^. Equation 7 linearly gives the Langmuir form^63^.

7 $$\:\frac{{C}_{e}}{{q}_{e}}\:=\frac{1}{{q}_{max}{K}_{L}}\:+\:\frac{{C}_{e}}{{q}_{max}}$$

8 $$R_{L}=\frac{1}{{1+C}_{0}{K}_{L}}$$

9 $$\:\text{ln}{q}_{e}=\:\text{ln}{K}_{F}\:+\:\frac{1}{n}\text{ln}{C}_{e}$$

C_e_, q_e_, q_max_, and K_L_ denote the dye concentration in equilibrium, the adsorption amount at equilibrium, the maximum adsorption capacity the model theoretically estimates, and the Langmuir constant. Equation 8 establishes the type of isotherm model based on the separation factor (R_L_). The classifications are as follows: favorable for 0 < R_L_ < 1, unfavorable for R_L_ > 1, reversible for R_L_ = 1, and irreversible for R_L_ = 0. The starting concentration of the dye is denoted by C_0_ (mg/L). The Freundlich isotherm model posits that adsorbate molecules accumulate on the adsorbent surface in multiple layers. The Freundlich isotherm model is shown in Eq. 9 in its linear form. In this case, n is the adsorption intensity, and K_F_ indicates the Freundlich constant (L/mg). The adsorption is uniform, favorable, or unfavorable at 1/*n* = 1, 1/*n*<1, and 1/*n*>1. The values of R_L_, q_max_, K_L_, 1/n, K_F,_ and R^2^ are summarized in Table 3. The most appropriate isotherm model is chosen based on the R^2^ value. The high R² value of 0.992 indicates that the Langmuir model is the most appropriate and the nature of the MO adsorption process over 10% PPy/C film is monolayer adsorption. The R_L_ value demonstrated favorable MO adsorption onto the 10% PPy/C. In addition the calculated q_max_ (~ 217 mg/g) from Langmuir model is near to the experimental quantity adsorbed (~ 203 mg/g).

**Table 3:** The isotherms (Langmuir and Freundlich) and kinetics (PFO and PSO) parameters for the sorption of MO dye onto 10% PPy/C film.

**Isotherm Models**						
**Langmuir**				**Freundlich**		
R^2^	q_max_ (mg/g)	K_L_ (L/mg)	R_L_	R^2^	K_f_ (L/mg)	1/n
0.992	217.3	0.7	0.017	0.94	86.48	0.345
**Kinetic Models**						
** Pseudo-first order (PFO)**			** Pseudo-second order (PSO)**			
R^2^	K_1_	qe (mg/g)	R^2^	K_2_	qe (mg/g)	
0.982	0.0256	163.04	0.999	0.0002	173.4	

### Dye adsorption kinetic studies

**Fig. 11:**
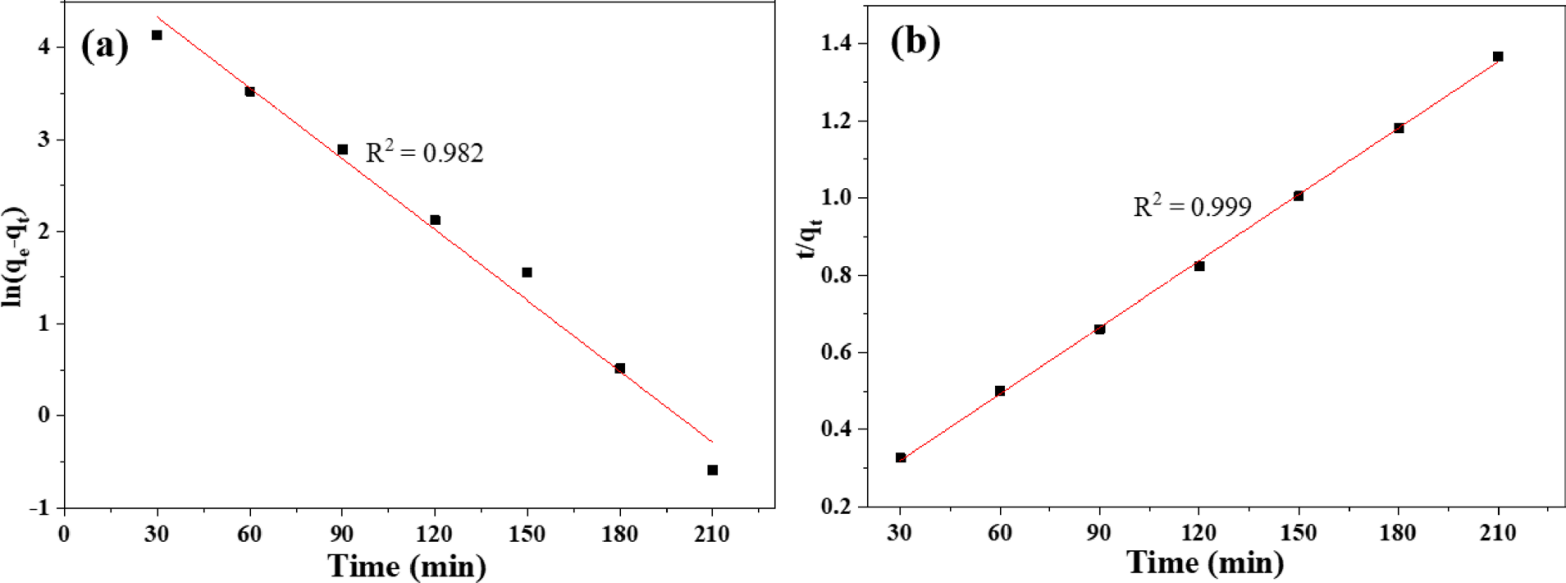
The fitting of (**a**) Pseudo first order (PFO) and (**b**) Pseudo second order (PFO) kinetic models for adsorption of MO dye using 10% PPy/C. The points and line represent the experimental and linear fitting, respectively.

The pseudo-second-order (PSO) and pseudo-first-order (PFO) models were applied to the experimental data regarding dye adsorption onto the 10% PPy/C film, which were conducted using a dose of 0.5 g/L and 80 ppm of MO dye. Figure 11a, b show PFO and PSO kinetic model fitting, respectively. The physisorption process and adsorption rate are assumed to decrease linearly with increasing adsorption capacity in the PFO kinetic model. The PSO model assumes that the adsorption process has been described as chemisorption^63^. The magnitude of the correlation coefficient (R^2^) is used to measure the model’s suitability to represent the adsorption performance. The values of R^2^ and the parameters of the kinetic model are detailed in Table 3. Equations 10 and 11 present the PFO and PSO models^63^.

10 $$\:\text{Ln}\left({q}_{e}-{q}_{t}\right)=\:\text{ln}{q}_{e}-{K}_{1}t$$

11 $$\:\frac{t}{{q}_{t}}\:=\:\frac{1}{{K}_{2}{q}_{e}^{2}}\:+\:\frac{1}{{q}_{e}}t$$

In this context, q_e_ and q_t_ represent the adsorption capacity at equilibrium and time t in mg/g, respectively. K_1_ (min^−1^) denotes the pseudo-first-order rate constant, while K_2_ (g/mg.min) signifies the pseudo-second-order adsorption rate constant.

Table 3 shows the R^2^, estimated q_e_, K_1_, K_2_, and experimental qe values. By comparing the calculated R^2^ values, the PSO is the most fitted one to the experimental sorption results of MO onto the 10% PPy/C. These data proved that the MO adsorption onto 10% PPy/C film is a pseudo-second-order process, and the rate-controlling step is the chemisorption step. The estimated q_max_ is 173.4 mg/g.

### The possible adsorption mechanisms and reusability

To gain a better understanding of how the adsorption process occurred, the charge on the surface of the synthesized 10% PPy/C film was assessed by using the pH-drift technique to determine the pH_ZPC_ (pH for the point of zero charge), as previously presented^68^, to discuss the possible MO adsorption mechanism. The surface is neutral at pH_PZC_; below this point, the surface charge is positive and negative above this threshold. Briefly, the pH values of different 0.1 M NaCl solutions were altered to pH values ranging from 10 to 4 using 0.1 M NaOH or HCl (assigned as pH_i_). Then, 50 mg of the film was added to 50 mL of the NaCl solution and agitated at room temperature. After 48 h, the final pH was measured (assigned as pH_f_) to calculate ΔpH (ΔpH = pH_f_ ‒ pH_i_). Finally, the pH_i_ was drawn vs.ΔpH to determine the pH at which ΔpH = 0 (i.e. the surface is uncharged), which is the pH_ZPC_^69^. The estimated pH_ZPC_ of 10% PPy/C was 8.3. Therefore, the film is positively charged in MO solution with pH ~ 8. Consequently, the film exhibited enhanced dye removal properties up to 95%. On the other hand, at pH = 10, the film surface is negatively charged; thus, the adsorption of anionic MO dye declined. This agrees with the experimental data for the impact of pH on dye removal.

**Scheme 2:**
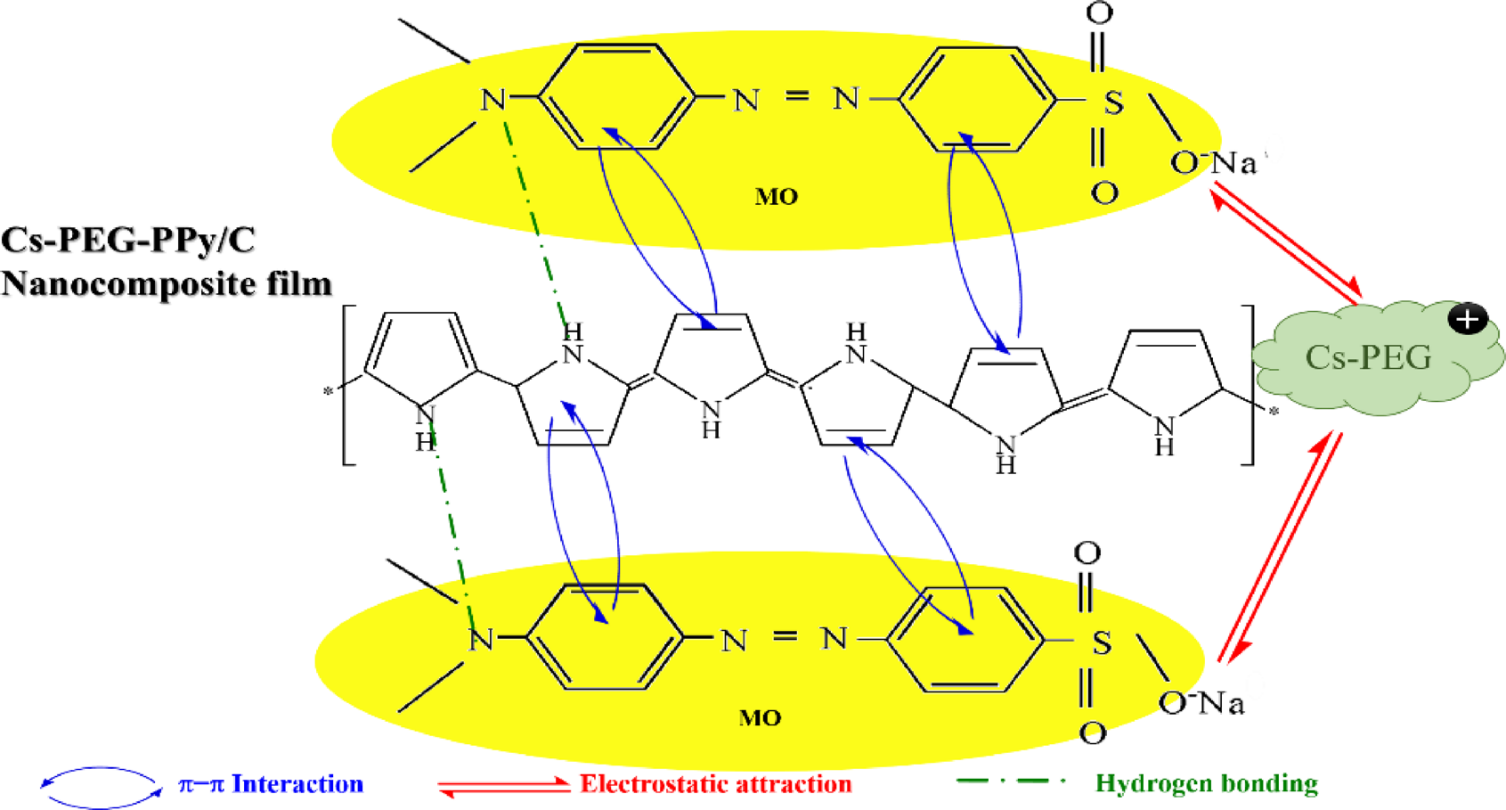
Schematic of the possible adsorption mechanism for MO onto PPy/C nanocomposite film.

The possible adsorption mechanisms of MO dye onto the PPy/C nanocomposite film can be due to three interactions, including π-π interaction, hydrogen bonding, and electrostatic interaction, as shown in Scheme 2. Briefly, the aromatic ring of PPy in the nanocomposite film may interact with the benzene ring of MO dye to form the π-π interaction^70,71^. There is an electrostatic interaction between the negatively charged sulfonate group of MO and the protonated amine groups of the Cs/PEG polymer blend^72^. The nitrogen atom of the MO dye (C-N- group) can form hydrogen bonds with the PPy/C backbone^73^.

After four consecutive adsorption/desorption cycles, the dye removal was 85%. The blockage of adsorbents’ active areas can be the reason for the decrease in removal efficiency. Finally, the produced adsorbent has practical and feasible uses in the removal of MO from water. NaOH was used to desorb MO from 10% PPy/C film.

### Comparative study

The adsorption amount of the developed 10% PPy/C film for MO dye, compared to kinetic-based capacity values for the previously reported materials, is listed in Table 4. These adsorbents were selected based on their structural similarity and the kinetic-based sorption capacity values. Table 4 shows that the prepared film is comparable in performance to different adsorbents. Specifically, the sorption capacities of MO onto the surface of N-containing polymers, i.e., polypyrrole^74^ and polyaniline^75^, were 147 and 111 mg/g, respectively, and are less than that for the synthesized 10% PPy/C film. Furthermore, the film exhibited higher dye adsorption than commercial activated carbon and lower contact time^76^. This may be attributed to the synergistic effect of PPy/C filler and chitosan/PEG polymer-making material.

**Table 4:** Comparison of the maximal monolayer adsorption capacity of 10% PPy/C film with other adsorbents utilized for MO dye adsorption.

Adsorbent	Q_max_ (mg/g)	Sorption conditions: C_0_ (mg/L), Dosage (D, g/L), Contact Time (t, min), pH	References
Commercial activated carbon	96	C_0_ = 80, D = 0.75, t = 250, pH = 2	^76^
Non-doped mesoporous carbons	120	C_0_ = 200, D = 1.0, t = 90, without any pH adjustment	^77^
Nitrogen-doped mesoporous carbons	135	C_0_ = 200, D = 1.0, t = 90, without any pH adjustment	^77^
Polypyrrole	147	C_0_ = 150, D = 1.0, t = 120, pH = 7	^74^
Polyaniline	111	C_0_ = 120, D = 1.0, t = 20, pH = 7	^75^
Poly(aniline-co-pyrrole)-based activated carbon	182	C_0_ = 200, D = 0.16, t = 60, pH = 6.6	^78^
Polypyrrole-based activated carbon	204	C_0_ = 200, D = 0.16, t = 60, pH = 6.6	^78^
10% PPy/C film	217	C_0_ = 120, D = 0.5, t = 260, pH = 8	The present study

## Conclusions

This study presents the successful synthesis and characterization of novel conductive (CS/PEG)-(PPy/C) composite films for potential application in dye removal. The integration of PPy/C into the Cs/PEG matrix markedly affected the composite films’ structural, morphological, optical, and electrical characteristics. XRD and FTIR analyses confirmed the successful integration of PPy/C into the CS/PEG blend, while FESEM images revealed the formation of agglomerated spherical micro-nano particles within the polymer matrix. The optical properties of the composite films showed enhanced reflectance and refractive index values with increasing PPy/C content, indicating their potential for optical confinement applications. Electrical studies demonstrated improved dielectric constant and AC conductivity with the addition of PPy/C, which was assigned to the films’ internal conductive network development. The AC conduction mechanism was discovered following the correlated barrier hopping (CBH) model. Important factors influencing the dyes’ adsorption onto the composites, like PPy/C amount, contact time, initial MO concentration, adsorbent dosage, and initial pH, were investigated. The 10% PPy/C composite film showed the best removal effectiveness relative to other films. The.

isotherm and kinetic study showed that the isotherm and kinetic data best fit to Langmuir and pseudo-second-order models, respectively. These indicate the spontaneous monolayer adsorption and the chemisorption-dominated mechanism. The maximum adsorption capacity of 217 mg/g for MO dye suggests the efficacy of the composite film in dye removal applications. In conclusion, this study presents a promising approach for developing conductive polymer composite films with improved electrical properties and efficient dye removal capabilities. The (CS/PEG)-(PPy/C) composite films offer a potential solution for addressing water pollution issues, particularly in treating dye-contaminated effluents from various industries.

## Data Availability

The datasets used and/or analyzed during the current study are available from the corresponding author upon reasonable request.
